# Evolution of CD4 T-Cell Count With Age in a Cohort of Young People Growing Up With Perinatally Acquired Human Immunodeficiency Virus

**DOI:** 10.1093/cid/ciad626

**Published:** 2023-10-11

**Authors:** Hannah Castro, Caroline Sabin, Intira Jeannie Collins, Hajra Okhai, Katrine Schou Sandgaard, Katia Prime, Caroline Foster, Marthe Le Prevost, Siobhan Crichton, Nigel Klein, Ali Judd, Collaborative HIV Paediatric Study, Hermione Lyall, Alasdair Bamford, Karina Butler, Katja Doerholt, Conor Doherty, Caroline Foster, Julia Kenny, Nigel Klein, Gillian Letting, Paddy McMaster, Fungai Murau, Edith Nsangi, Katia Prime, Andrew Riordan, Fiona Shackley, Delane Shingadia, Sharon Storey, Gareth Tudor-Williams, Anna Turkova, Steve Welch, Intira Jeannie Collins, Claire Cook, Siobhan Crichton, Donna Dobson, Keith Fairbrother, Diana M Gibb, Ali Judd, Marthe Le Prevost, Nadine Van Looy, Helen Peters, Kate Francis, Claire Thorne, L Thrasyvoulou, S Welch, K Fidler, J Bernatoniene, F Manyika, G Sharpe, B Subramaniam, R Hague, V Price, J Flynn, A Cardoso, M Abou–Rayyah, N Klein, A Bamford, D Shingadia, K Grant, S Yeadon, S Segal, S Hawkins, M Dowie, S Bandi, E Percival, M Eisenhut, K Duncan, L Anguvaa, L Wren, T Flood, A Pickering, P McMaster, C Murphy, J Daniels, Y Lees, F Thompson, A Williams, B Williams, S Pope, S Libeschutz, L Cliffe, S Southall, A Freeman, H Freeman, S Christie, A Gordon, L Jones, L Brown, M Greenberg, C Benson, A Riordan, L Ibberson, F Shackley, S Patel, J Hancock, K Doerholt, K Prime, M Sharland, S Storey, E G H Lyall, C Foster, P Seery, G Tudor-Williams, N Kirkhope, S Raghunanan, Dr J Kenny, A Callaghan, A Bridgwood, P McMaster, J Evans, E Blake, A Yannoulias, UK Collaborative HIV Cohort Study, Jonathan Ainsworth, Sris Allan, Jane Anderson, Ade Apoola, David Chadwick, Duncan Churchill, Valerie Delpech, David Dunn, Ian Fairley, Ashini Fox, Richard Gilson, Mark Gompels, Phillip Hay, Rajesh Hembrom, Teresa Hill, Margaret Johnson, Sophie Jose, Stephen Kegg, Clifford Leen, Dushyant Mital, Mark Nelson, Hajra Okhai, Chloe Orkin, Adrian Palfreeman, Andrew Phillips, Deenan Pillay, Ashley Price, Frank Post, Jillian Pritchard, Caroline Sabin, Achim Schwenk, Anjum Tariq, Roy Trevelion, Andy Ustianowski, John Walsh, David Dunn, Teresa Hill, Hajra Okhai, Andrew Phillips, Caroline Sabin, Nadine van Looy, Keith Fairbrother, Chloe Orkin, Janet Lynch, James Hand, Duncan Churchill, Stuart Tilbury, Elaney Youssef, Duncan Churchill, Mark Nelson, Richard Daly, David Asboe, Sundhiya Mandalia, Jane Anderson, Sajid Munshi, Frank Post, Ade Adefisan, Chris Taylor, Zachary Gleisner, Fowzia Ibrahim, Lucy Campbell, David Chadwick, Kirsty Baillie, Richard Gilson, Ian Williams, Jonathan Ainsworth, Achim Schwenk, Sheila Miller, Chris Wood, Margaret Johnson, Mike Youle, Fiona Lampe, Colette Smith, Rob Tsintas, Clinton Chaloner, Caroline Sabin, Andrew Phillips, Teresa Hill, Hajra Okhai, John Walsh, Nicky Mackie, Alan Winston, Jonathan Weber, Farhan Ramzan, Mark Carder, Clifford Leen, Andrew Kerr, David Wilks, Sheila Morris, Mark Gompels, Sue Allan, Adrian Palfreeman, Adam Lewszuk, Stephen Kegg, Victoria Ogunbiyi, Sue Mitchell, Phillip Hay, Christopher Hunt, Olanike Okolo, Benjamin Watt, Ian Fairley, Sarah Russell-Sharpe, Olatunde Fagbayimu, Sris Allan, Debra Brain, Anjum Tariq, Liz Radford, Sarah Milgate, Jillian Pritchard, Shirley Cumming, Claire Atkinson, Dushyant Mital, Annie Rose, Jeanette Smith, Andy Ustianowski, Cynthia Murphy, Ilise Gunder, Ashini Fox, Howard Gees, Gemma Squires, Laura Anderson, Rajesh Hembrom, Serena Mansfield, Lee Tomlinson, Christine LeHegerat, Roberta Box, Tom Hatton, Doreen Herbert, Ashley Price, Ian McVittie, Victoria Murtha, Laura Shewan, Ade Apoola, Zak Connan, Luke Gregory, Kathleen Holding, Victoria Chester, Trusha Mistry, Catherine Gatford, Valerie Delpech, Roy Trevelion

**Affiliations:** Institute of Clinical Trials and Methodology, Medical Research Council Clinical Trials Unit at University College London, University College London, London, United Kingdom; Institute for Global Health, University College London, London, United Kingdom; National Institute for Health and Care Research, Health Protection Research Unit in Blood Borne and Sexually Transmitted Infections at University Colllege London, University College London, London, United Kingdom; Institute of Clinical Trials and Methodology, Medical Research Council Clinical Trials Unit at University College London, University College London, London, United Kingdom; Institute for Global Health, University College London, London, United Kingdom; Department of Pediatrics and Adolescent Medicine, Aarhus University Hospital, Aarhus, Denmark; Department of Genitourinary Medicine, St George’s University Hospitals National Health Service Foundation Trust, London, United Kingdom; Department of Paediatric Infectious DIseases, Imperial College Healthcare National Health Service Trust, London, United Kingdom; Institute of Clinical Trials and Methodology, Medical Research Council Clinical Trials Unit at University College London, University College London, London, United Kingdom; Institute of Clinical Trials and Methodology, Medical Research Council Clinical Trials Unit at University College London, University College London, London, United Kingdom; Infection, Immunity and Inflammation, University College London, Great Ormond Street Institute of Child Health, London, United Kingdom; Institute of Clinical Trials and Methodology, Medical Research Council Clinical Trials Unit at University College London, University College London, London, United Kingdom

**Keywords:** CD4 T cell, perinatal, HIV, child, adult

## Abstract

**Background:**

Recent studies have shown a decrease in CD4 count during adolescence in young people with perinatally acquired human immunodeficiency virus (HIV, PHIV).

**Methods:**

Young people with PHIV in the United Kingdom, followed in the Collaborative HIV Paediatric Study who started antiretroviral therapy (ART) from 2000 onward were included. Changes in CD4 count over time from age 10 to 20 years were analyzed using mixed-effects models, and were compared to published CD4 data for the gerneral population. Potential predictors were examined and included demographics, age at ART start, nadir CD4 *z* score (age-adjusted) in childhood, and time-updated viral load.

**Results:**

Of 1258 young people with PHIV included, 669 (53%) were female, median age at ART initiation was 8.3 years, and the median nadir CD4 *z* score was −4.0. Mean CD4 count was higher in young people with PHIV who started ART before age 10 years and had a nadir CD4 *z* score ≥−4; these young people with PHIV had a decline in CD4 count after age 10 that was comparable to that of the general population. Mean CD4 count was lower in young people with PHIV who had started ART before age 10 and had a nadir CD4 *z* score <−4; for this group, the decline in CD4 count after age 10 was steeper over time.

**Conclusions:**

In children, in addition to starting ART at an early age, optimizing ART to maintain a higher CD4 *z* score during childhood may be important to maximizing immune reconstitution later in life.

## Teaser

Young people with human immunodeficiency virus who started antiretroviral therapy before age 10 years and had a nadir CD4 z score <-4 in childhood, had a decline in CD4 count after age 10 steeper than that of the general population.

## Graphical Abstract

**Graphical Abstract:**
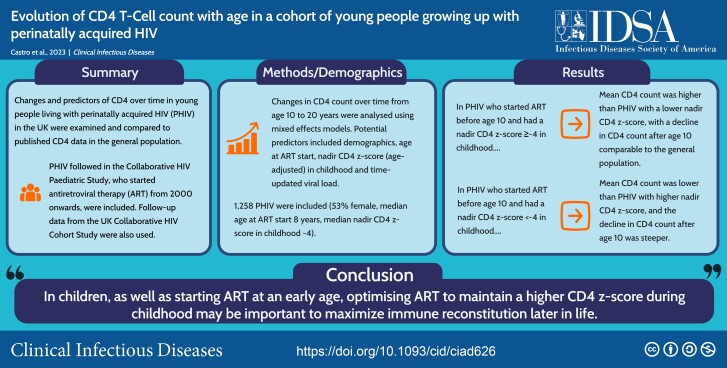
This graphical abstract is also available at Tidbit: https://tidbitapp.io/tidbits/evolution-of-cd4-t-cell-count-with-age-in-a-cohort-of-young-people-growing-up-with-perinatally-acquired-hiv-e628fcfc-4e7a-4d1f-b878-ebf12b31f134

In 2021, there were an estimated 1.7 million adolescents with human immunodeficiency virus (HIV) worldwide [1]. Due to the availability of effective antiretroviral therapy (ART), children living with perinatally acquired HIV (PHIV) are surviving into adulthood [2]. However, some studies have shown poor health outcomes for young people with PHIV compared with younger children and adults with HIV, including lower levels of virological suppression, worse immunological status, poor growth, and lower retention in care [3–7].

Current guidelines state that ART should be initiated immediately for all individuals with HIV [8], and initial CD4 recovery following the start of ART in children has been well described [9]. The few studies that have examined long-term CD4 evolution have shown a decrease in CD4 count during adolescence [7, 10, 11]. However, the reasons for this decline remain unclear, including how much is part of the natural decline in CD4 count seen after infancy in the general population [12]. Previous studies have predicted that children who start ART at an early age are likely to follow a trajectory of CD4 that is expected in the general population [13, 14], and higher CD4 counts before/at ART initiation have been associated with higher long-term CD4 [13–16] and a better CD4 response after treatment interruptions [17].

Our aim in this analysis was to examine changes in and predictors of CD4 over time in young people with PHIV in the United Kingdom and to compare the changes to published data on CD4 in young people in the general population. A previous analysis specifically explored CD4 changes before and after transition to adult care in young people who had been followed in the Collaborative HIV Paediatric Study (CHIPS) and subsequently transitioned to adult clinics in the UK Collaborative HIV Cohort (UK CHIC) Study [10] and did not compare the results to the general population. Here, we included all young people followed in CHIPS and linked updated datasets from the 2 studies, resulting in a larger cohort of young people with PHIV and with longer follow-up through pediatric and adult HIV care than previously.

## METHODS

CHIPS is a national observational cohort of children with HIV in the United Kingdom and Ireland [18]. In brief, all infants born to women with HIV and all children aged <16 years at HIV diagnosis in the United Kingdom and Ireland are reported to the Integrated Screening Outcomes Surveillance Service (formerly the National Study of HIV in Pregnancy and Childhood). Children diagnosed with HIV were reported to CHIPS and followed from first presentation in pediatric care until the child transitioned to adult care. Follow-up data were collected annually until March 2021. The UK CHIC Study was a multicenter study of adults with HIV aged ≥16 years attending 1 of 21 collaborating adult outpatient clinics across the United Kingdom [19]. Routinely collected clinical information was submitted annually (until September 2019) from participating clinics to the study database. Both studies had National Health Service Research Ethics approval.

In this analysis, young people followed in CHIPS with documented PHIV who started ART during or after 2000 with at least 1 CD4 measurement at age ≥10 years were included. They were linked to the UK CHIC database using their date of birth and sex as well as Soundex (an indexing system that encodes surnames), clinic name/clinic number, or the young person’s initials. Data used for the linkage were stored in a secure location with restricted access. Young people were grouped by age at the start of ART and nadir CD4 *z* score (age-adjusted using CD4 counts) in childhood [20] to create 6 groups (A: started ART at age ≤5 years and nadir CD4 *z* score <−4, B: started ART at age ≤5 years and nadir CD4 *z* score ≥−4, C: started ART at age >5 to <10 years and nadir CD4 *z* score <−4, D: started ART at age >5 to <10 years and nadir CD4 *z* score ≥−4, E: started ART at age ≥10 years and nadir CD4 *z* score <−4, and F: started ART at age ≥10 years and nadir CD4 *z* score ≥−4). Nadir CD4 *z* score in childhood was defined as the lowest CD4 *z* score before age 10; for young people without CD4 measurements before age 10, it was defined as the CD4 *z* score at the start of ART (closest measurement within 6 months of starting ART).

Data were analyzed using Stata version 17.0 (Stata Corp, College Station, TX). Characteristics of the young people included in the analysis with non-missing values were summarized using proportions, medians, and interquartile range (IQR). Data were missing for <10% of young people for each variable unless specified. Immunodeficiency was categorized based on the World Health Organization definition for children aged >5 years using CD4 count [21].

Changes in CD4 count over time were analyzed using mixed-effects models, allowing for multiple CD4 measurements per young person. All available CD4 measurements from CHIPS and UK CHIC were analyzed from age 10 years to age 20 years. Time since age 10 was modeled using both linear and quadratic variables that were included in all models. Person-level random effects were included for intercept and slopes (unstructured covariance matrix). The effect of predictors of changes in CD4 over time was explored and included the following variables: sex, ethnicity, country of birth, year of birth, age at ART start and nadir CD4 *z* score groups, transitioned to adolescent/adult care, and suppressed viral load <400 copies/mL within 6 months of the CD4 measurement (time-updated); ART regimen was not included as a predictor. Variables were included in the multivariable model using backward elimination, and a *P* value <.05 was considered statistically significant. Interactions between time/time squared since age 10 and each variable were added to the multivariable model if the interaction *P* value was <.05. The statistical significance of predictors in the multivariate model are reported in the results section as *P* values adjusted for the presence of the other variables in the final model. Due to changes in the frequency of CD4 measurements over time, a variable for time since previous CD4 measurement (≤4 months vs >4 months) was included in a sensitivity analysis. Additional sensitivity analyses included fitting the multivariable model separately to young people linked and not linked to UK CHIC.

Published data on CD4 counts in young people aged 10 to 20 years in the general population were identified using PubMed. Relevant search terms included lymphocyte, subset/subpopulation, reference/control value/range, child/childhood, and adolescent. Reference and cited-by lists from all articles found to be relevant were also searched.

## RESULTS

Of 1258 young people with PHIV included in the analysis, 53% were female and 84% were of Black ethnicity (Table 1). The median age at ART initiation was 8.3 years, and the median nadir CD4 *z* score in childhood was −4.0. Most young people had no or mild immunodeficiency at age 10 (88%). Only 66% (612 of 932) of young people with available data had a suppressed viral load <400 copies/mL at age 10 (592 of 693 [85%] for those who started ART before age 10). At last follow-up, 797 (63%) had transitioned to adolescent/adult care and 464 (37%) were linked to UK CHIC. Of those not linked, 389 (49%) were still being followed in CHIPS, 355 (45%) had transitioned to an adult clinic not participating in UK CHIC, 40 (5%) were lost to follow-up, and 10 (1%) had died. Twenty-four percent (304) had ever had an AIDS-defining diagnosis; of those, 75 had their first AIDS event after age 10, 59 of whom had started ART aged ≥10. Sixteen (1%) had died at a median age of 19 years (2 within 1 year of diagnosis), with 13 starting ART at age ≥10.

**Table 1.** Characteristics of the 1258 Young People Included in the Analysis

	Total (n = 1258)	Linked to UK Collaborative HIV Cohort (n = 464)
Characteristic	n (%) or Median (IQR)	
Female sex	669 (53)	245 (53)
Black ethnicity	1047 (84)	396 (86)
Born outside United Kingdom/Ireland	775 (62)	312 (67)
Year of birth		
Up to 1996	381 (30)	233 (50)
1997 to 2000	429 (34)	208 (45)
2001 onward	448 (36)	23 (5)
Year of starting ART		
2000 to 2004	442 (35)	210 (45)
2005 to 2009	468 (37)	165 (36)
2010 onward	348 (28)	89 (19)
Age started ART, y	8.3 (3.5 to 12.1)	10.3 (6.5 to 13.2)
≤5	406 (32)	83 (18)
>5 and <10	352 (28)	140 (30)
≥10	500 (40)	241 (52)
Nadir CD4 *z* score^a^	−4.0 (−5.9 to −2.5)	−4.3 (−6.8 to −2.8)
<−4	588 (49)	237 (54)
≥−4	602 (51)	202 (46)
Age started ART (y) and nadir CD4 *z* score^a^		
A: Started ART ≤5 y of age and nadir CD4 *z* score <−4	138 (12)	34 (8)
B: Started ART ≤5 y of age and nadir CD4 *z* score ≥−4	259 (22)	46 (10)
C: Started ART >5 to <10 y of age and nadir CD4 *z* score <−4	211 (18)	89 (20)
D: Started ART >5 to <10 y of age and nadir CD4 *z* score ≥−4	115 (10)	44 (10)
E: Started ART ≥10 y of age and nadir CD4 *z* score <−4	239 (20)	114 (26)
F: Started ART ≥10 y of age and nadir CD4 *z* score ≥−4	228 (19)	112 (26)
Median (IQR) age (y) started ART within age started ART and nadir CD4 *z* score groups^a^		
A: Started ART ≤5 y of age and nadir CD4 *z* score <−4	2.5 (0.7 to 3.8)	3.4 (2.4 to 4.0)
B: Started ART ≤5 y of age and nadir CD4 *z* score ≥−4	1.2 (0.3 to 2.8)	2.4 (1.6 to 3.4)
C: Started ART >5 to <10 y of age and nadir CD4 *z* score <−4	7.9 (6.8 to 8.9)	8.2 (7.3 to 9.1)
D: Started ART >5 to <10 y of age and nadir CD4 *z* score ≥−4	6.9 (5.8 to 8.0)	6.9 (5.9 to 7.8)
E: Started ART ≥10 y of age and nadir CD4 *z* score <−4	12.6 (11.3 to 14.2)	12.5 (11.2 to 14.2)
F: Started ART ≥10 y of age and nadir CD4 *z* score ≥−4	13.1 (11.6 to 14.8)	13.7 (12.0 to 15.6)
CD4 count at age 10 y (cells/mm^3^, within ±6 m)^b^	724 (480 to 998)	621 (405 to 910)
Immunodeficiency at age 10 y (within ±6 m)^b^		
None or not significant (≥500 cells/mm^3^)	685 (73)	207 (63)
Mild (≥350 to <500 cells/mm^3^)	144 (15)	65 (20)
Advanced (≥200 to <350 cells/mm^3^)	86 (9)	39 (12)
Severe (<200 cells/mm^3^)	28 (3)	17 (5)
CD4 *z* score at age 10 y (within ±6 m)^b^	−1.3 (−2.9 to −0.2)	−1.9 (−3.6 to −0.5)
Viral load <400 copies/mL at age 10 y (within ±6 m)^c^	612 (66)	174 (54)
Known to have transitioned to adolescent/adult care	797 (63)	442 (95)
Ever CDC class C (AIDS) diagnosis	304 (24)	115 (25)
Ever CDC class C (AIDS) at age 10 y	228 (18)	70 (15)
First CDC class C (AIDS) event after age 10 y^d^	75 (6)	44 (9)
A: Started ART ≤5 y of age and nadir CD4 *z* score <−4	2 (0.2)	1 (0.2)
B: Started ART ≤5 y of age and nadir CD4 *z* score ≥−4	1 (0.1)	1 (0.2)
C: Started ART >5 to <10 y of age and nadir CD4 *z* score <−4	2 (0.2)	1 (0.2)
D: Started ART >5 to <10 y of age and nadir CD4 *z* score ≥−4	3 (0.3)	3 (0.7)
E: Started ART ≥10 y of age and nadir CD4 *z* score <−4	38 (3)	21 (5)
F: Started ART ≥10 y of age and nadir CD4 *z* score ≥−4	21 (2)	13 (3)
Age at first CDC class C (AIDS) event (y)	3.4 (0.6 to 10.0)	7.8 (2.7 to 12.4)
Died^e^	16 (1)	6 (1)
C: Started ART >5 to <10 y of age and nadir CD4 *z* score <−4	1 (0.1)	…
D: Started ART >5 to <10 y of age and nadir CD4 *z* score ≥−4	1(0.1)	…
E: Started ART ≥10 y of age and nadir CD4 *z* score <−4	8 (0.7)	4 (0.9)
F: Started ART ≥10 y of age and nadir CD4 *z* score ≥−4	5 (0.4)	2 (0.4)
Age when died, y	18.8 (15.2 to 22.7)	22.7 (20.3 to 25.2)
Years since diagnosis when died, y	10.6 (6.0 to 14.8)	11.7 (10.0 to 14.8)

Abbreviations: AIDS, acquired immunodeficiency syndrome; ART, antiretroviral therapy; CDC, Centers for Disease Control and Prevention; HIV, human immunodeficiency virus; IQR, interquartile range.

^a^Nadir CD4 *z* score was defined as the lowest CD4 *z* score before age 10 years (n = 945); for young people without CD4 measurements before age 10, nadir CD4 *z* score was defined as CD4 *z* score at start of ART (within 6 months; n = 245). Unknown for 68 young people.

^b^Unknown for 315 young people.

^c^Unknown for 326 young people.

^d^Nadir CD4 *z* score unknown for 8 young people with first CDC class C (AIDS) event after age 10 years.

^e^All died after age 10. Nadir CD4 *z* score unknown for 1 young person who died.

A total of 26 270 CD4 measurements from 1996 to 2020 were available for analysis. Young people had a median (IQR) of 2.8 (2.1 to 3.3) CD4 measurements per year (only 24 [2%] had only 1 CD4 measurement), and the median age at the last CD4 measurement was 17.8 years (IQR, 16.0 to 19.3). Of the 258 young people who were immunosuppressed (<500 cells/mm^3^) at age 10, 42 of 63 (67%) with a CD4 measurement at age 20 (within ±6 months) were still immunosuppressed at age 20. In multivariable analysis, mean CD4 count at age 10 differed by age at the start of ART and nadir CD4 *z* score, with young people who had started ART at a younger age and had a higher nadir CD4 *z* score in childhood having higher CD4 counts at age 10 (*P* < .001; Table 2). Young people who were female (*P* < .001), of non-Black ethnicity (*P* = .002), born in later calendar years (*P* < .001), and virally suppressed (*P* < .001) also had higher mean CD4 counts at age 10.

**Table 2.** Univariate and Multivariable Predictors of CD4 Count Over Time From Age 10 Years to Age 20 Years

	Univariable^a^			Multivariable (n = 1181)		
Predictor	Coefficient	95% CI	*P* Value	Coefficient	95% CI	*P* Value
Constant	…	…	…	664.1	588.2 to 739.9	…
Main effects	…	…	…	…	…	…
Time since age 10 (per 1-y increase after age 10)	−13.1	−16.3 to −9.9	<.001	1.4	−10.8 to 13.6	.824
Time since age 10 squared (per year squared increase after age 10)	−1.7	−2.1 to −1.4	<.001	−3.4	−4.6 to −2.2	<.001
Female (vs male)	21.6	−7.9 to 51.1	.151	67.9	31.2 to 104.5	<.001
Non-Black ethnicity (vs Black ethnicity)	96.5	56.5 to 136.6	<.001	52.3	18.8 to 85.8	.002
Born abroad (vs born in United Kingdom/Ireland)	−54.1	−84.6 to −23.6	.001	…	…	…
Year of birth (per 1-y increase since 1985)	23.5	20.4 to 26.6	<.001	6.7	3.4 to 10.0	<.001
Age started ART (y) and nadir CD4 *z* score	…	…	<.001	…	…	<.001
A: Started ART ≤5 y of age and nadir CD4 *z* score <−4	0.0	…	…	0.0	…	…
B: Started ART ≤5 y of age and nadir CD4 *z* score ≥−4	165.6	117.5 to 213.8	…	197.3	132.5 to 262.0	…
C: Started ART >5 to <10 y of age and nadir CD4 *z* score <−4	−94.0	−143.1 to −44.8	…	−97.7	−165.0 to −30.4	…
D: Started ART >5 to <10 y of age and nadir CD4 *z* score ≥−4	44.2	−13.3 to 101.7	…	132.4	54.9 to 209.9	…
E: Started ART ≥10 y of age and nadir CD4 *z* score <−4	−264.8	−312.7 to −216.9	…	−533.6	−604.8 to −462.4	…
F: Started ART ≥10 y of age and nadir CD4 *z* score ≥−4	−95.4	−143.7 to −47.1	…	−137.6	−206.8 to −68.4	…
Known to have transitioned to adolescent/adult care (vs not known to have transitioned)	−129.6	−160.6 to −98.7	<.001	…	…	…
Viral suppression (time updated, within ±6 m of CD4 count)	…	…	<.001	…	…	<.001
Suppressed, <400 copies/mL	0.0	…	…	0.0	…	…
Not suppressed, ≥400 copies/mL	−149.8	−156.1 to −143.6	…	−78.6	−96.5 to −60.7	…
Interactions with time/time squared since age 10	…	…	…	…	…	…
Sex (time)	…	…	…	…	…	<.001
Male	…	…	…	0.0	…	…
Female	…	…	…	−21.6	−29.9 to −13.4	…
Sex (time squared)	…	…	…	…	…	<.001
Male	…	…	…	0.0	…	…
Female	…	…	…	1.9	1.2 to 2.7	…
Age started ART (y) and nadir CD4 *z* score (time)	…	…	…	…	…	<.001
A: Started ART ≤5 y of age and nadir CD4 *z* score <−4	…	…	…	0.0	…	…
B: Started ART ≤5 y of age and nadir CD4 *z* score ≥−4	…	…	…	−33.5	−48.8 to −18.3	…
C: Started ART >5 to <10 y of age and nadir CD4 *z* score <−4	…	…	…	3.8	−10.8 to 18.3	…
D: Started ART >5 to <10 y of age and nadir CD4 *z* score ≥−4	…	…	…	−35.7	−53.0 to −18.3	…
E: Started ART ≥10 y of age and nadir CD4 *z* score <−4	…	…	…	102.8	86.9 to 118.7	…
F: Started ART ≥10 y of age and nadir CD4 *z* score ≥−4	…	…	…	18.0	2.5 to 33.5	…
Age started ART (y) and nadir CD4 *z* score (time squared)	…	…	…	…	…	<.001
A: Started ART ≤5 y of age and nadir CD4 *z* score <−4	…	…	…	0.0	…	…
B: Started ART ≤5 y of age and nadir CD4 *z* score ≥−4	…	…	…	3.5	2.0 to 5.1	…
C: Started ART >5 to <10 y of age and nadir CD4 *z* score <−4	…	…	…	0.9	−0.5 to 2.3	…
D: Started ART >5 to <10 y of age and nadir CD4 *z* score ≥−4	…	…	…	3.5	1.8 to 5.1	…
E: Started ART ≥10 y of age and nadir CD4 *z* score <−4	…	…	…	−4.7	−6.1 to −3.2	…
F: Started ART ≥10 y of age and nadir CD4 *z* score ≥−4	…	…	…	1.4	0.0 to 2.9	…
Viral suppression (time updated, within ±6 m of CD4 count) (time)	…	…	…	…	…	<.001
Suppressed, <400 copies/mL	…	…	…	0.0	…	…
Not suppressed, ≥400 copies/mL	…	…	…	−26.5	−34.4 to −18.6	…
Viral suppression (time updated, within ±6 m of CD4 count) (time squared)	…	…	…	…	…	<.001
Suppressed, <400 copies/mL	…	…	…	0.0	…	…
Not suppressed, ≥400 copies/mL	…	…	…	2.1	1.3 to 2.9	…

Abbreviations: ART, antiretroviral therapy; CI, confidence interval.

^a^Number of young people in the univariable models, n = 1258; except for ethnicity, n = 1249; country of birth, n = 1256; years on ART at age 10 and nadir CD4 *z* score, n = 1190; and viral suppression, n = 1257.

**Figure 1.**
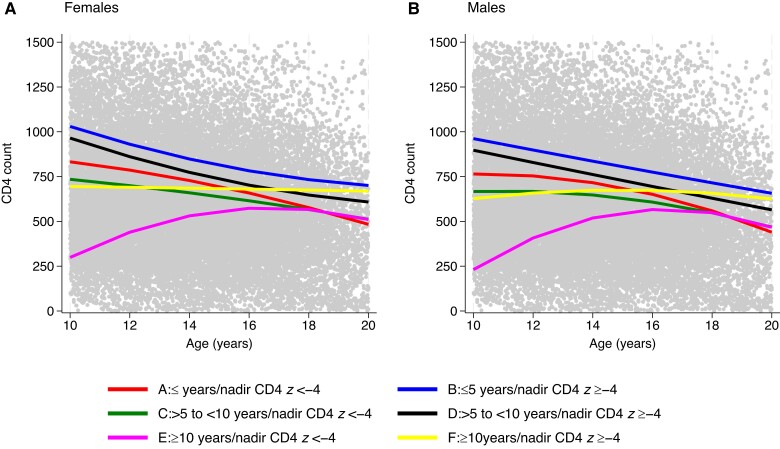
Predicted mean CD4 counts over time for young people with perinatal human immunodeficiency virus of Black ethnicity born in 2000 with suppressed viral load (time updated) by age at the start of antiretroviral therapy/nadir CD4 *z* score groups.

In multivariable analysis, CD4 counts over time also differed by age at the start of ART and nadir CD4 *z* score and by sex (*P* < .001 for all time variables; Table 2). Figure 1 shows the predicted association between CD4 over time (age 10 to 20 years) and age at the start of ART/nadir CD4 *z* score groups for modeled females (Figure 1*A*) and males (Figure 1*B*) of Black ethnicity and born in 2000 with a suppressed viral load <400 copies/mL (time-updated). At age 10, mean CD4 count was highest in young people who had started ART before age 10 and had a higher nadir CD4 *z* score, with a decline in CD4 count after age 10 that slowed over time in females (Figure 1*A*, groups B and D; Supplementary Table 1*A*) and was more linear in males (Figure 1*B*, groups B and D; Supplementary Table 1*B*). Mean CD4 count at age 10 was lower in young people who had started ART before age 10 and had a lower nadir CD4 *z* score (Figure 1*A* and 1*B*, groups A and C; Supplementary Table 1*A* and 1*B*); the decline in CD4 over time was steeper than in those who had a higher nadir CD4 *z* score (groups B and D).

At age 10, young people who had started ART at age ≥10 years and had a higher nadir CD4 *z* score (group F) had a mean CD4 count that was similar to that of group C; however, the CD4 count remained stable in group F over time with a predicted average of 650 cells/mm^3^ for both males and females (Figure 1*B*; Supplementary Table 1*A* and 1*B*). At age 10, mean CD4 count was lowest in young people who had started ART at age ≥10 and had a lower nadir CD4 *z* score (group E). In this group, CD4 increased over time until approximately age 16 years, plateaued, and then decreased, with a similar trend in males and females.

There was no evidence that mean CD4 counts differed over time by ethnicity or year of birth. On average, being virally unsuppressed was associated with having greater decreases or smaller increases in CD4 over time than being suppressed (*P* < .001). There was no evidence of an effect of time since last CD4 measurement on CD4 trends over time (*P* = .168), and fitting separate multivariable models to young people linked and not linked to UK CHIC found comparable results to the overall multivariable model (data not shown).

Published data from 9 studies (7 in Europe or the United States) of CD4 counts in the general population were identified [22–30] (Table 3). All studies were in young people without any known medical conditions except for 1 study that included young people admitted to the hospital for presurgery screenings for malformations, trauma, or benign diseases [24] and 1 that included young people with bleeding disorders but no lymphocyte abnormalities [25].

**Table 3.** Published Data on CD4 Count in Young People in the General Population

					Average^a^ CD4 Count (cells/mm^3^) At Age (y)					
Study	Country	Age Range	Ethnicity	Gender	10	12	14	16	18	20
Comans-Bitter et al [22], 1997	Netherlands	0 to adults 5–10 y; n = 35 10–16 y; n = 23 adults; n = 51	NG	NG	1000 (300–2000)^b^	800 (400–2100)^b^			700 (300–1400)^b^	
Huenecke et al [23], 2008	Germany	2 m to 40 y 4–10 y; n = 31 10–18 y; n = 10 adults; n = 20	NG	35% female	986^c^ (499–1588)	954^c^ (483–1537)	939^c^ (475–1512)	931^c^ (471–1500)	928^c^ (469–1494)	926^c^ (468–1491)
Tosato et al [24], 2015	Italy	0 to 18 y 6–12 y; n = 56 12–18 y; n = 20	85% White	42% female	1030 (646–1515)		887 (610–1446)			…
Bofill et al [25], 1992	United Kingdom	1 to 79 y 9–10 y; n* = 25 11–79 y; n* = 600	NG	NG	980^d^ (243)	830^d^ (288)				
Valiathian et al [26], 2014	United States	12 to 67 y 12–18 y; n = 50	NG	61% female	…	920 (467–1563)^e^				…
Shearer et al [27], 2003	United States	0 to 18 y 6–12 y; n = 90 12–18 y; n = 90	53% African American	48% female	980 (650–1500)		840 (530–1300)			…
Rudy et al [28], 2002	United States	14 to 20 y 14 y; n* = 3(m), 5(f) 16 y; n* = 13(m),55(f) 18 y; n* = 36(m), 108(f) 20 y; n* = 11(m),35(f)	63% African American	77% female	…	…	m: 833^d^ (143) f: 779^d^ (243)	m: 632^d^ (107) f: 866^d^ (287)	m: 753^d^ (207) f: 817^d^ (273)	m: 712^d^ (192) f: 881^d^ (319)
Mandala et al [29], 2010	Malawi	0 to 92 y 5–10 y; n = 52 10–15 y; n = 49 15–20 y; n = 51	African	53% female	1200 (800–2100)	1100 (800–1700)		900 (600–1200)		
de Moraes-Pinot et al [30], 2014	Brazil	0 to 49 y 6–12 y; n = 50 12–18 y; n = 50 19–48 y; n = 51	NG	43% female	858 (566–1298)		847 (640–1279)			813 (487–1141)

Abbreviations: f, female; m, male; n, number of participants (except for * where n is number of tests (number of participants was not given); NG, not given.

^a^Median and 10th and 90th percentiles in parenthesis, unless otherwise stated.

^b^5th and 95th percentiles.

^c^Predicted values from exponential model and 90% lower and upper bounds in parenthesis.

^d^Mean and standard deviation in parenthesis.

^e^Range.

**Figure 2.**
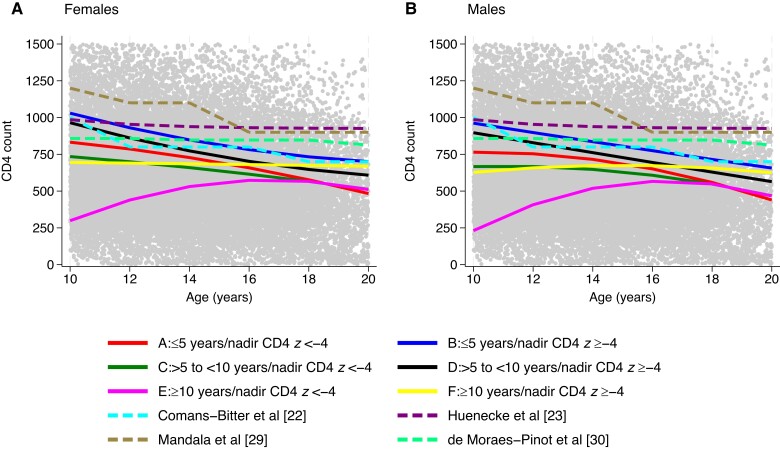
Predicted mean CD4 counts over time for young people with perinatal human immunodeficiency virus of Black ethnicity born in 2000 with suppressed viral load (time-updated) by age at the start of antiretroviral therapy/nadir CD4 *z* score groups compared with published data on CD4 count in young people in the general population.

Figure 2 shows published data from 4 studies that included young people aged 10 to 20 years and had 3 or more age groups in that range [22, 23, 29, 30]. At age 10, the predicted mean CD4 count in young people in our study who started ART before age 10 and had a higher nadir CD4 *z* score (groups B and D) was similar to CD4 counts in young people in studies from the Netherlands (Comans–Bitter et al [22]) and Germany (Huenecke et al [23]), with the predicted decline in CD4 over time in groups B and D comparable to the Comans-Bitter study but faster than the Huenecke study. By age 20 years, young people who had a lower nadir CD4 *z* score, regardless of the age at which they started ART (groups A, C, and E), had a predicted mean CD4 count lower than that observed in all 4 studies of young people in the general population.

## DISCUSSION

We found that changes in CD4 count over time in a cohort of young people with PHIV varied by age at ART start and nadir CD4 *z* score in childhood. For young people who started ART before the age of 10, CD4 count decreased from age 10. For those with a higher nadir CD4 *z* score, the predicted decline in some subgroups was comparable to published data of CD4 counts in young people in the general population. However, for those who had a lower nadir CD4 *z* score, the decline was steeper, and results from our model, assuming, on average, viral suppression <400 copies/mL over time, predicted average CD4 counts to approach mild immunodeficiency (350–500 cells/mm^3^) by age 20 years in some subgroups with characteristics associated with lower mean CD4 counts.

In our previous study, we found a decline in CD4 count over time in 271 young people in the period before transition to adult care that continued after transition in some groups. However, we were unable to assess the trends by age at ART start and nadir CD4 due to limited sample size [10]. This current analysis builds on our previous work and highlights the long-term impact of these factors and how the CD4 trajectory compares to the general population in Europe.

Rodriguez et al [11] found that in 132 young people who started ART at a median age of 5.7 years in Peru, CD4 count (unadjusted for viral load) decreased from age 5 to 18 years and that the decline was faster after age 13 years. A global cohort collaboration [7] also found that CD4 count (unadjusted for viral load) decreased from age 10 to 17 years in 19 557 young people with PHIV who started ART at a median age of 6.9 years in 46 countries worldwide, with similar trends by sex and geographical region.

The decline in CD4 count observed in our study in young people who started ART before the age of 5 years and had a higher nadir CD4 *z* score in childhood appears to mirror the decline in CD4 observed in least 1 study in young people in the general population. However, due to the small sample size, there is large variation in the median estimate. Mean CD4 counts also followed a similar trajectory over time for young people who started ART at ages between 5 and 10 years and had a higher nadir CD4 *z* score; at age 20, mean CD4 counts were predicted to be higher than for those who had a lower nadir CD4 *z* score and started ART at any age.

For young people who started ART before age 10 and had a lower nadir CD4 *z* score in childhood, the decline in CD4 was steeper. Children with HIV have a profound capacity for immune recovery following ART, as thymic output is high in infancy [31]. However, our study implies that immune reconstitution may not only rely on early age of ART initiation but also on the immune system’s ability to maintain a normal level of CD4 T-cell numbers during childhood. Previous studies have suggested the importance of a high thymic output and diverse T-cell receptor repertoire in maintaining viral suppression as well as contributing to high CD4 later in life [32, 33]. It is reassuring that there were few AIDS events or deaths in young people who started ART before age 10 in our study. However, future research is needed to determine whether the steeper decline in CD4 counts seen in young people with a lower nadir CD4 during childhood results in different clinical outcomes during adulthood compared with the general population.

We found that changes in CD4 count over time differed by sex, with females having higher mean CD4 counts than males in early and late adolescence. Previous studies in children [34, 35] and adults [36, 37] have suggested that females have a better immunological response than males after ART initiation, and Rudy et al [28] found that females in the general population without HIV infection had higher CD4 counts than males.

One limitation of our study is that we were unable to examine the effects of adherence to ART on CD4 evolution, as adherence information was not available. However, we adjusted our analysis for time-updated viral load as a surrogate. Another limitation is that we did not adjust for ART regimen, and young people who started ART in earlier calendar years and on older regimens may have contributed more CD4 data over time than those on newer regimens. However, we included only young people who started ART from 2000 onward, when effective ART was available for children. Also, findings from young people in our study who started ART at older ages may not be relevant to infants starting ART today. Last, young people lost to follow-up or who died before age 10 would not have contributed to the analysis, and some young people in CHIPS transitioned to non-UK CHIC clinics and would not have contributed CD4 data after transition. Young people participating in CHIPS had high retention in pediatric care and low mortality rates [3], and sensitivity analyses fitting the model to young people linked and not linked to UK CHIC found comparable results.

We have shown that for young people with PHIV and who started ART before age 10, having a lower nadir CD4 *z* score in childhood was associated with a decline in CD4 count over time. This suggests that in children, in addition to starting ART at an early age, optimizing ART to maintain good levels of immune function may be important to maximize immune reconstitution later in life. Young people who start ART before age 10 with a higher nadir CD4 *z* score during childhood are likely to achieve CD4 levels during adolescence and early adulthood that are comparable to those of young people in the general population.

## Supplementary Data

Supplementary materials are available at *Clinical Infectious Diseases* online. Consisting of data provided by the authors to benefit the reader, the posted materials are not copyedited and are the sole responsibility of the authors, so questions or comments should be addressed to the corresponding author.

## Supplementary Material

ciad626_Supplementary_Data

## Notes

***Author Contributions*.** H. C., N. K., C. S., A. J., and H.O. substantially contributed to the concept and design of the study. All authors substantially contributed to the acquisition, analysis, or interpretation of the data. S. C., M. L. P., and H. O. acquired and verified the data. H. C. performed the analysis. H. C., N. K., C. S., A. J., I. J. C., S. C., K. S. S., K. P., and C. F. interpreted the data. H. C. drafted the article, with major contributions from C. S., A. J., I. J. C., S. C., and K. S. S. All authors revised the manuscript for important intellectual content, approved the final version for publication, and are accountable for all aspects of the work.
