# Supplementary material for: Evolution of CD4 T-Cell Count With Age in a Cohort of Young People Growing Up With Perinatally Acquired Human Immunodeficiency Virus
Source: Clin Infect Dis. 2023 Oct 11;78(3):690–701. doi: 10.1093/cid/ciad626 (PMC10954325; doi:10.1093/cid/ciad626)
Supplement: ciad626_Supplementary_Data [file ciad626_supplementary_data.zip › Supplementary Table 1a_140723_1.3 (CID).docx]

**Supplementary Table 1a: Predicted mean CD4 counts [95% confidence interval] over time for females with perinatal HIV, of black ethnicity, born in 2000 with suppressed viral load (time updated), by age at the start of ART/nadir CD4 z-score groups.**

| Age (years): | 10 | 12 | 14 | 16 | 18 | 20 |
| --- | --- | --- | --- | --- | --- | --- |
| A: Started ART age ≤5 years/ nadir CD4 z-score <-4 | 832 [776, 889] | 786 [741, 832] | 728 [688, 769] | 658 [617, 700] | 577 [525, 628] | 483 [410, 557] |
| B: Started ART age ≤5 years/ nadir CD4 z-score ≥-4 | 1030 [985, 1074] | 931 [895, 967] | 848 [815, 881] | 782 [748, 817] | 733 [688, 778] | 700 [634, 767] |
| C: Started ART age >5 to <10 years/  nadir CD4 z-score <-4 | 735 [689, 780] | 700 [663, 736] | 660 [627, 692] | 615 [582, 648] | 566 [526, 605] | 512 [458, 565] |
| D: Started ART age >5 to <10 years/ nadir CD4 z-score ≥-4 | 965 [906, 1024] | 861 [814, 908] | 774 [731, 816] | 702 [658, 746] | 647 [592, 702] | 608 [532, 684] |
| E: Started ART age ≥10 years/ nadir CD4 z-score <-4 | 299 [248, 350] | 439 [401, 478] | 531 [497, 565] | 573 [540, 607] | 566 [527, 605] | 511 [459, 562] |
| F: Started ART age ≥10 years/ nadir CD4 z-score ≥-4 | 695 [648, 742] | 690 [654, 726] | 685 [654, 717] | 680 [649, 712] | 675 [638, 712] | 669 [618, 720] |

Abbreviations: ART, antiretroviral therapy
