# Supplementary material for: Evolution of CD4 T-Cell Count With Age in a Cohort of Young People Growing Up With Perinatally Acquired Human Immunodeficiency Virus
Source: Clin Infect Dis. 2023 Oct 11;78(3):690–701. doi: 10.1093/cid/ciad626 (PMC10954325; doi:10.1093/cid/ciad626)
Supplement: ciad626_Supplementary_Data [file ciad626_supplementary_data.zip › Supplementary Table 1b_140723_1.3 (CID).docx]

**Supplementary Table 1b: Predicted mean CD4 counts [95% confidence interval] over time for males with perinatal HIV, of black ethnicity, born in 2000 with suppressed viral load (time updated), by age at the start of ART/nadir CD4 z-score groups.**

| Age (years): | 10 | 12 | 14 | 16 | 18 | 20 |
| --- | --- | --- | --- | --- | --- | --- |
| A: Started ART age ≤5 years/ nadir CD4 z-score <-4 | 765 [710, 819] | 754 [710, 798] | 716 [677, 755] | 651 [611, 691] | 559 [510, 608] | 440 [369, 510] |
| B: Started ART age ≤5 years/ nadir CD4 z-score ≥-4 | 962 [917, 1007] | 898 [862, 934] | 836 [803, 869] | 775 [741, 809] | 715 [671, 760] | 657 [591, 722] |
| C: Started ART age >5 to <10 years/ nadir CD4 z-score <-4 | 667 [620, 713] | 667 [630, 704] | 648 [615, 681] | 608 [574, 641] | 548 [508, 588] | 468 [414, 523] |
| D: Started ART age >5 to <10 years/ nadir CD4 z-score ≥-4 | 897 [835, 959] | 829 [780, 878] | 761 [718, 805] | 695 [650, 740] | 629 [574, 685] | 564 [487, 642] |
| E: Started ART age ≥10 years/ nadir CD4 z-score <-4 | 231 [180, 282] | 407 [368, 446] | 519 [485, 553] | 566 [532, 600] | 549 [510, 588] | 467 [416, 519] |
| F: Started ART age ≥10 years/ nadir CD4 z-score ≥-4 | 627 [576, 678] | 658 [619, 697] | 673 [639, 707] | 673 [639, 707] | 657 [616, 698] | 625 [570, 680] |

Abbreviations: ART, antiretroviral therapy
